# Supplementary material for: Preoperative and operation-related risk factors for postoperative nosocomial infections in pediatric patients: A retrospective cohort study
Source: PLoS One. 2019 Dec 23;14(12):e0225607. doi: 10.1371/journal.pone.0225607 (PMC6927644; doi:10.1371/journal.pone.0225607)
Supplement: S1 File — (DOC) [file pone.0225607.s001.doc]

**S1 File**

**Table A. Coding for the candidate risk factors**

| Risk factor | Coding |
| --- | --- |
| Sex | 0=female, 1=Male |
| Age | Continuous |
| Indicator of WAZ | 0=missing/NA, 1=available |
| WAZ | Continuous |
| Days of preoperative hospitalization | Continuous |
| Indicator of preoperative ICU stay | 0=no, 1=yes |
| Days in ICU | 0 if the indicator=0  Days in ICU – 1 if the indicator=1 |
| Preoperative EN | 0=no, 1=yes |
| Preoperative PN | 0=no, 1=yes |
| Preoperative antibiotic prophylaxis | 0=no, 1=yes |
| Indicator of preoperative blood test | 0=no, 1=yes |
| Hemoglobin (unit=5g/L) | 0 if the indicator=0  Hemoglobin – 20 if the indicator=1 |
| WBC (unit=5×10^9^/L) | 0 if the indicator=0  WBC − 2 if the indicator=1 |
| Surgical implantation | 0=no, 1=yes |
| Indicator of operative duration | 0=missing, 1=available |
| Operative duration (unit=1 hour) | 0 if the indicator=0  Operative duration − 1 if the indicator=1 |
| Indictor of ASA score | 0=no/missing, 1=yes |
| ASA score | 0 if the indicator=0  0=I, 1=II, 2=III/IV/V, if the indicator=1 |
| Indicator of SWC | 0=no surgical incision, 1=surgical incision |
| SWC | 0 if the indicator=0  0=clean, 1=clean-contaminated, 2=contaminated, if the indicator=1 |

ASA (American Society of Anesthesiologists), EN (enteral nutrition), ICU (intensive care unit), NA (not applicable), PN (parenteral nutrition), SWC (surgical wound classification), WAZ (weight-for-age z-score), WBC (white blood cell).

**Table B. Associations between baseline factors and the development of postoperative NIs in multivariable Cox models, the Guangzhou Women and Children’s Medical Center, 2016–2018**

|  | Full model^a^ | | |  | Reduced model^a^ | | |
| --- | --- | --- | --- | --- | --- | --- | --- |
|  | HR | 95% CI | *P* |  | HR | 95% CI | *P* |
| Sex, male *vs.* female | 0.93 | 0.81–1.08 | 0.34 |  |  |  |  |
| Age, per year increase | 0.95^b^ | 0.92–0.98 | < 0.01 |  | 0.95^b^ | 0.92–0.98 | < 0.01 |
| WAZ |  |  |  |  |  |  |  |
| Indicator, yes *vs.* NA/missing | 0.74 | 0.49–1.10 | 0.14 |  | 0.73 | 0.49–1.09 | 0.13 |
| Per unit increase | 0.94 | 0.90–0.99 | 0.03 |  | 0.95 | 0.90–1.00 | 0.03 |
| Preoperative hospitalization |  |  |  |  |  |  |  |
| Per day increase | 1.03 | 1.02–1.04 | < 0.01 |  | 1.03 | 1.02–1.04 | < 0.01 |
| Preoperative ICU stay |  |  |  |  |  |  |  |
| Indicator, yes *vs.* no | 1.02 | 0.87–1.20 | 0.78 |  | 1.01 | 0.86–1.18 | 0.91 |
| Per day increase | 0.98 | 0.96–0.99 | < 0.01 |  | 0.98 | 0.96–0.99 | < 0.01 |
| Preoperative EN, yes *vs.* no | 0.83 | 0.69–0.99 | 0.04 |  | 0.80 | 0.67–0.96 | 0.02 |
| Preoperative PN, yes *vs.* no | 0.89 | 0.71–1.10 | 0.28 |  |  |  |  |
| Antibiotic prophylaxis |  |  |  |  |  |  |  |
| Yes *vs.* no | 0.70 | 0.60–0.83 | < 0.01 |  | 0.72^b^ | 0.61–0.85 | < 0.01 |
| Preoperative blood test |  |  |  |  |  |  |  |
| Indicator, yes *vs.* no | 1.30 | 1.03–1.65 | 0.03 |  | 1.30 | 1.03–1.64 | 0.03 |
| Hemoglobin, per 5 g/L increase | 0.97 | 0.95–0.98 | < 0.01 |  | 0.97 | 0.95–0.98 | < 0.01 |
| WBC, per 5×10^9^/L increase | 1.02 | 1.00–1.03 | 0.02 |  | 1.02 | 1.00-1.03 | 0.02 |
| Surgical implantation, yes *vs.* no | 1.16 | 0.93–1.43 | 0.18 |  |  |  |  |
| Operative duration |  |  |  |  |  |  |  |
| Indicator, yes *vs.* missing | 0.47^b^ | 0.35–0.62 | < 0.01 |  | 0.47^b^ | 0.35–0.63 | < 0.01 |
| Per hour increase | 1.14 ^b^ | 1.08–1.19 | < 0.01 |  | 1.14^b^ | 1.08–1.20 | < 0.01 |
| ASA score, |  |  |  |  |  |  |  |
| Indicator, yes *vs.* no | 1.32 | 0.91–1.92 | 0.14 |  | 1.32 | 0.91–1.92 | 0.14 |
| ASA score II *vs.* I | 1.05 | 0.80–1.36 | 0.74 |  | 1.05 | 0.80–1.36 | 0.74 |
| ASA score ≥III *vs.* I | 1.41 | 1.06–1.89 | 0.02 |  | 1.43 | 1.07–1.91 | 0.02 |
| SWC, |  |  |  |  |  |  |  |
| Indicator, yes *vs.* no | 0.96 ^b^ | 0.80–1.15 | 0.66 |  |  |  |  |
| Clean-contaminated *vs.* clean | 1.05 | 0.77–1.43 | 0.76 |  |  |  |  |
| Contaminated *vs.* clean | 1.21 | 0.76–1.93 | 0.41 |  |  |  |  |

^a^Both models were adjusted for operative site. ^b^Proportional hazards assumption was violated. ASA (American Society of Anesthesiologists), EN (enteral nutrition), HR (hazard ratio), ICD-9-CM (International Classification of Diseases, 9^th^ Revision, Clinical Modification), ICU (intensive care unit), NA (not applicable), PN (parenteral nutrition), SWC (surgical wound classification), WAZ (weight-for-age z-score), WBC (white blood cell).

**Risk score calculation**

A risk score (RS) weighted by the reduced AFT model’s coefficients was calculated for each patient using the following formula:

RS=0.0641×age.surg+0.2701×WAZ.indicator + 0.0452×WAZ–0.0318×presurg.hdays +0.0381×presurgICU.indicator + 0.0264× (presurgICU.days–1) + 0.2177*presurg.EN + 0.6999×opdur.indicator – 0.1025×(opedur – 60) – 0.3962×ASA.indicator – 0.0244×(ASA=II) – 0.238×(ASA=III/IV/V) + 0.3526×presurg.antibio – 0.1974*presurg.bldtest.indcator+ 0.0303×(presurg.hb/5–20) – 0.0142×(presurg.wbc/5 –2).

In the formula:

age.surg: age at surgery

WAZ.indicator: indicator for WAZ

presurg.hdays: days of hospital stay before surgery

presurgICU.indicator: indicator for ICU stay before surgery

presurgICU.days: days of ICU stay before surgery

presurg.EN: enteral nutrition before surgery

opdur.indicator: indicator for operation duration

opedur: duration of operation (in minutes)

ASA.indicator: indicator for ASA score

presurg.antibio: use of antibiotics before surgery

presurg.bldtest.indcator: indicator for blood test before surgery

presurg.hb: hemoglobin level before surgery

presurg.wbc: white blood cell count before surgery
