# Supplementary material for: Preoperative and operation-related risk factors for postoperative nosocomial infections in pediatric patients: A retrospective cohort study
Source: PLoS One. 2019 Dec 23;14(12):e0225607. doi: 10.1371/journal.pone.0225607 (PMC6927644; doi:10.1371/journal.pone.0225607)
Supplement: Attachment — Submitted filename: PlosReview.pdf [file pone.0225607.s002.pdf]

Manuscript Overview

This manuscript provides a retrospective analysis of nosocomial infections in pediatric patients undergoing invasive operations.

Major Comments

1. The statistical inference lacks scientific context beyond stating statistical significance. The American Statistical Association's statement on p-values highlights this point: *A p-value, or statistical significance, does not measure the size of an effect or the importance of a result.* For instance, line 269 states "We found that longer preoperative ICU stay was associated with delayed occurrence of postoperative NIs:..." How influential was this in terms of the occurrence probability? The entire discussion lacks this effect size discussion.

2. L 109 - 111. What are the implications of only the first invasive operation / postoperative NI being analyzed? Does this result in any bias? Is it expected that follow up surgeries would have different rates of NI occurrence? My understanding is that if the first invasive operation did not result in a NI, then this data point would be included and all later invasive operations would be excluded. Is this correct?

3. L 139 - 140. Be specific about how the variables were treated as 'missing'. Missing data is a loaded word with a whole subfield of statistics devoted to it. How does the missing data and associated binary indicator impact the statistical models?

4. L 148 - 149. How do the standard discharge rates (days after surgery) differ between the surgery types? Figure 1 shows the risk up to 7 days post operation. How large would the sample sizes be for 7 - days post operation for all surgery types? I'm curious if the analysis with incidence rate per patient days is a good comparison if many (most!) patients of a certain surgery type are released from the hospital shortly after surgery. How is this treated in the statistical models? Is this type of analysis standard, if so include supporting evidence/citations, otherwise, would an analysis of likelihood of experiencing an NI for a given surgery be appropriate?

5. How are the ICD-9-CM codes used in the statistical models? Table 2 only states both models were adjusted for operative site - how so? Are these intercept values or would a more general model (such as a hierarchical model) that allows different responses to explanatory variables across the groups be more appropriate?

6. Line 234. How are the high-risk and low-risk groups defined? I don't see this in the text, but the caption for Figure 2 states, "median of the risk score derived from the reduced AFT model". Why is this grouping useful or interesting? It is not surprising that the high-risk group would have higher NI incidence rates, but this does suggest the overall model is doing something useful.

Minor Comments

- Use commas to separate five or more numbers, such as 18314 → and 18,314.
- L 80, From prevention perspective → from a prevention perspective.
- L 155 - 159. It is not clear how the block structures works in practice. Do the p-values for all variables in a block need to be under the specified threshold?

- P 9. “averagely” could be reworded/replaced.
- Table 1 contains a lot of information. One thing that might help would be lines or something to signify sub-categories (within SWC and ASA score).
- Table 2, typo “paediatric”
- Table 3. Is the full model necessary?
