# Supplementary material for: Preoperative and operation-related risk factors for postoperative nosocomial infections in pediatric patients: A retrospective cohort study
Source: PLoS One. 2019 Dec 23;14(12):e0225607. doi: 10.1371/journal.pone.0225607 (PMC6927644; doi:10.1371/journal.pone.0225607)
Supplement: Attachment — Submitted filename: Rebuttal Letter.pdf [file pone.0225607.s003.pdf]

Point-to-Point Response

Reviewer #1: The paper is well written, methods appear appropriate, and the results provide useful information on risk factors for post-operative infection following paediatric surgery. The reference list needs tidying for consistent formatting.

In the revision, we have formatted the reference list following the Plos ONE style.

Reviewer #2: This manuscript provides a retrospective analysis of nosocomial infections in pediatric patients undergoing invasive operations.

Major Comments:

- The statistical inference lacks scientific context beyond stating statistical significance. The American Statistical Association's statement on p-values highlights this point: *A p-value, or statistical significance, does not measure the size of an effect or the importance of a result*. For instance, line 269 states "We found that longer preoperative ICU stay was associated with delayed occurrence of postoperative NIs:...". How influential was this in terms of the occurrence probability? The entire discussion lacks this effect size discussion.

We agree with the reviewer that effect sizes are more relevant and informative measures than *P*-values. As we modeled the data with accelerated failure time regression, a more straightforward measure of the effect size is the number of days by which the factors could advance or delay the occurrence of the postoperative NIs. To calculate this quantity (page 7 line 155 - page 8 line 159), we defined a reference patient with a specific risk factor profile and provided information for some important risk factors regarding the reduced/increased days between the invasive operation and the occurrence of postoperative NIs (page 12 line 214-225). We also added the effect sizes for other risk factors across the Discussion section (page 16 line 2275- 277, line 287-288; page 17 line 306-307).

- L 109 - 111. What are the implications of only the first invasive operation / postoperative NI being analyzed? Does this result in any bias? Is it expected that follow up surgeries would have different rates of NI occurrence? My understanding is that if the first invasive operation did not result in a NI, then this data point would be included and all later invasive operations would be excluded. Is this correct?

Thank you for raising this important question. As we focused on the first invasive operation, it was likely that the model estimates, in particular the estimates for surgery-related characteristics, were biased because some of the NIs might be a result following the the second or the third surgeries. In our study, roughly 7% (1283/18314) of the patients underwent two or more invasive operations but the majority of the postoperative NIs (724/847, 85%) occurred before the second invasive operation, suggesting that the resulting bias might exist but we consider them be minor particularly for non-surgical factors such as nutritional status.

The NI rate following the second or the third surgeries is likely to be different from the NI rate after

the first surgery, but it is difficult to justify the direction of the change. In our study population, most of the patients received antibiotic treatment to prevent infection and some of them were given intensive care after the initial surgery, and these might affect their risk of developing NIs after the next surgery.

For the patients who remained NI-free during the entire study period (i.e. from the first invasive operation till hospital discharge), we censored them at the point of hospital discharge rather than at the point of the next invasive operation.

- L 139 - 140. Be specific about how the variables were treated as `missing'. Missing data is a loaded word with a whole subfield of statistics devoted to it. How does the missing data and associated binary indicator impact the statistical models?

In page 7 line 142 to 144, we described that for risk factors with some missing values, a binary indicator was created to denote data missingness, and this indicator variable was included into the model along with the original risk factors. Please note that the original risk factors were centered to a certain value (see the Appendix Table 1) to make the coefficient of the missingness indicator interpretable. For example, we introduced a binary indicator to denote missingness of operation duration and centered operation duration to 60 minutes if it was not missing. The coefficient of the binary indicator thus represents the effect size for patients with operation duration equal to 60 minutes versus patients whose operation duration was missing.

The impact of missing data on statistical models is a complex question without straightforward answers. However, in our case, if the analysis was limited to the patients with complete information for all covariates, we would lose more than 40% of the events. In addition, our results suggest that our data were not missing at random. Therefore we are convinced that the way we handled missing data would reduce the selection bias and increase the generalization of the results.

- L 148 - 149. How do the standard discharge rates (days after surgery) differ between the surgery types? Figure 1 shows the risk up to 7 days post operation. How large would the sample sizes be for 7 - days post operation for all surgery types? I'm curious if the analysis with incidence rate per patient days is a good comparison if many (most!) patients of a certain surgery type are released from the hospital shortly after surgery. How is this treated in the statistical models? Is this type of analysis standard, if so include supporting evidence/citations, otherwise, would an analysis of likelihood of experiencing an NI for a given surgery be appropriate?

In our patient population, the median of length of post-operation hospital stay was 13 days after hemic and lymphatic surgeries, 3 days after musculoskeletal surgery, and 7 days for the remaining surgeries. The table below gives the number of patients who stayed ≥ 7 days post operation.

Type of surgery	Staying ≥ 7 days post operation/all (%)
Nervous system	1253 /2353 (53.2)

Respiratory system	1029 /1875 (54.9)
Cardiovascular system	1719 /3233 (53.2)
Hemic and lymphatic system	356 /466 (76.4)
Digestive system	2912/5497 (53.0)
Urinary system	1511/2839 (53.2)
Musculoskeletal system	365/2051 (17.8)

The AFT model treated patients in two ways depending on their event status rather on, regardless of the length of post-operative hospital stay. As for patients who did not develop NI, they were kept in the model until discharge, while for those who developed NI, they were kept in the model until the time point when NI was diagnosed.

Incidence of NIs is typical time-to-event data and survival regression is a perfect tool for this type of data. Cox proportional hazards models have been widely used to analyze time-to-event data, but we have mentioned clearly that Cox model seemed not be a good choice for our data as the proportional hazards assumption was violated and thus we chose AFT models as a substitute (page 18 line 333-337).

- How are the ICD-9-CM codes used in the statistical models? Table 2 only states both models were adjusted for operative site - how so? Are these intercept values or would a more general model (such as a hierarchical model) that allows different responses to explanatory variables across the groups be more appropriate?

The ICD-9-CM code was introduced into the model as a covariate to control for the clinical heterogeneity (page 8 line 166-169). In the revision, we have further clarified this in the first place (page 7 line 137-138). In our opinion, this might not be a suitable scenario for hierarchical modeling, because the operative site measures the patient-level variation (such as sex), which is different from some extrinsic features (e.g. different hospitals from which the patients were recruited).

- Line 234. How are the high-risk and low-risk groups defined? I don't see this in the text, but the caption for Figure 2 states, "median of the risk score derived from the reduced AFT model". Why is this grouping useful or interesting? It is not surprising that the high-risk group would have higher NI incidence rates, but this does suggest the overall model is doing something useful.

Please refer to page 8 line 173 to page 9 line 179 for how the high-risk and low-risk groups were defined.

There is no doubt that a higher NI risk should be observed in the model-defined high-risk group and a lower risk in the low-risk group, as long as the model is not a complete failure, but it is necessary to quantify the difference, which reflects the model's discriminative capacity. Figure 2 exactly suggests how useful the model was for risk stratification by showing how apart the risk curve for the high-risk group and the risk curve for the low-risk group were separated from each other.

Minor Comments:

- Use commas to separate five or more numbers, such as 18314 -> and 18,314.

Done (page 2 line 38 and 39; page 6 line 114; page 10 line 194 and 196; page 14 line 235, 238, 239, 243, 244, and 246; Table 1; Table 2).

- L 80, From prevention perspective -> from a prevention perspective.

Thanks! The missing "a" has been added (page 4 line 80).

- L 155 - 159. It is not clear how the block structures works in practice. Do the p-values for all variables in a block need to be under the specified threshold?

No, variables in the same block were either all retained in or all excluded from the model depending on the Wald-test P value for the block as a whole. For example, the full model yielded a Wald statistic of 18 for the ASA block (i.e. the ASA indicator and the two ASA dummies combined), resulting in a P-value of 0.00044 under Chi-square distribution with df=3; therefore the ASA block should be retained in the model at least at this stage, and this test was repeated during the backward selection until the inclusion of the ASA block was finally determined.

- P 9. ``averagely'' could be reworded/replaced.

Thanks, the word has been replaced with "on average" (page 9 line 189).

- Table 1 contains a lot of information. One thing that might help would be lines or something to signify sub-categories (within SWC and ASA score).

We have indented the lines further to signify the sub-categories.

- Table 2, typo ``paediatric''

Corrected.

- Table 3. Is the full model necessary?

Yes, it was an important part of our analysis and some readers might be interested in it as well.

Reviewer #3: This review focuses on the *statistical aspects* of the submitted manuscript, exclusively.

In essence, the manuscript describes the statistical analysis of a hospital data-set containing pediatric peri-operative data and nosocomial infection status; this reviewer understands the goal of the study as identification of pre-operative and operation-related risk factors for NI.

The authors chose "time to event" as primary variable of interest, where event specifies either NI or discharge. (see major comment, below). The main model is "Accelerated failure time" (AFT). A (more standard, but measuring something slightly different) Cox model was used for comparison (but data only reported in appendix 2). The authors state that non-linear associations of continuous factors were considered, but none retained (if this reviewer understands correctly).

A reduced model was obtained by backward elimination. Identified time-ratios of factors were used to classify patients into low- and high-risk, respectively, with an enrichment of 70% of NI in the high-risk group.

The manuscript is well written, concise, and well-structured. There are a few items where this reviewer feels that the description should be more complete (verbose, but potentially also include a few math-stats expression for precision). Overall, this reviewer believes that with appropriate revisions the manuscript should be published (unless revisions bring major statistical flaws to light).

Comments that the authors should to address in revision:

1) major one: The authors are dealing with a highly censored data set. A majority of patients does not exhibit NI (or not on record, anyways). It is absolutely not clear to this reviewer, how the authors are handling censored events (i.e. distinguish time to event between NI and discharge). In particular since NI patients are in the minority, the main event is discharge---is the AFT model therefore primarily fitting time to discharge? It is this reviewer's strong belief that this "detail" should be described and discussed in much more detail, here. What are the exact assumptions? What does the AFT model look like (a mathematical expression or two would be appreciated)? How "log-normal" are the data (show it?)? How do NI events differ from discharge events? Is the AFT model fit to patients with NI, only, or the entire collection? etc.... Maybe the authors are doing everything correct, already, but from the description it is near impossible to know (or possible reproduce). This is the central element of the study and should be presented with sufficient detail (despite space limitations...).

We thank the reviewers for the positive comments as well as the questions.

In the initial submission, we stated that "Time to event was defined as the duration of date of operation to date of NI diagnosis or date of discharge, whichever came first" (page 7 line 149-150). For patients with NI, the time to event was the time between operation and NI diagnosis; for patients without NI, the time to event was the time between operation and discharge. These two types of time to event were distinguished using the indicator of event status (1 for NIs and 0 for non-NIs).

The assumption underlying the AFT model is that the log-transformed **time to develop NI** follows a normal distribution (in other words, the time to develop NI follows a log-normal distribution), despite that majority of the patients were censored at discharge. A general form of the AFT model is:

$$\log T_i = \mu + \beta_1 x_{1i} + \ldots + \beta_p x_{pi} + \sigma \varepsilon_i$$

However, like any other survival models, the AFT models utilized information from both patients with NIs and patients without NIs to build the likelihood function as follows:

$$L(\beta, \sigma; y_i) = \prod_{i=1}^{n} [f_Y(y_i)]^{\delta_i} [S_Y(y_i)]^{1-\delta_i}$$

where δ_i is the event indicator (1 if NI occurs, 0 if censored). This function indicates clearly that patients with and without NIs all contribute to the model: the former contribute in the form of their probability density function, while the latter contribute in the form of their survival function (or 1 minus cumulative density function).

The figure below shows the distribution of the time between invasive operation and postoperative NI diagnosis:

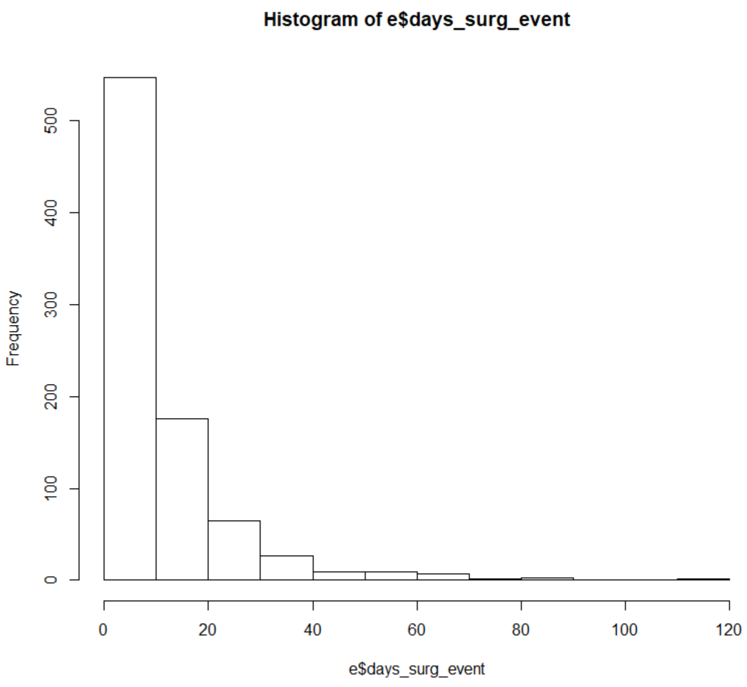

The distribution after log-transformation:

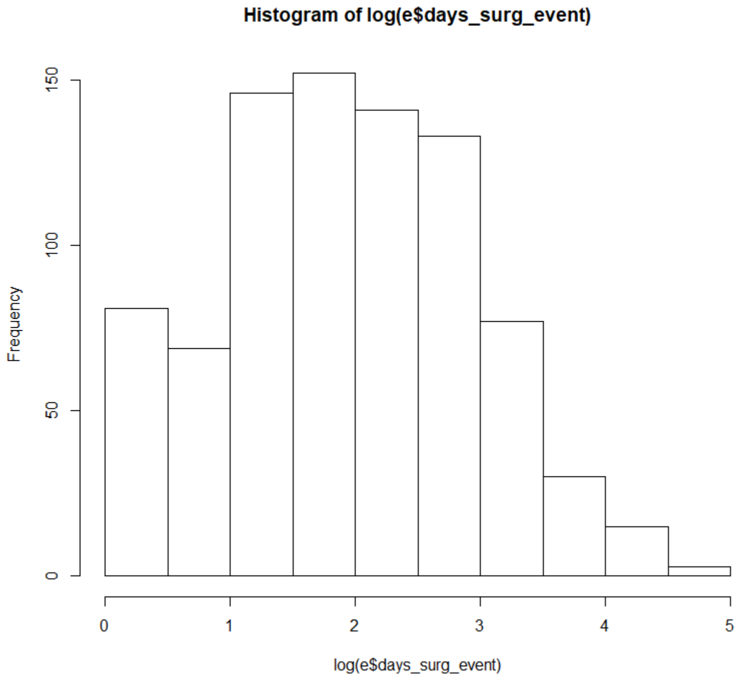

We hope the reviewer will agree with us that all the statistical details mentioned above should be left out of the manuscript as they might look too statistical for pediatricians and healthcare workers (to be frank, we ourselves are not statisticians).

2) The primary outcome of the AFT model is that certain factors accelerate or decelerate the progression of a patient through progress from operation to event (NI/discharge). How does this relate to NI *risk* (=what the cox model captures)? A description of the exact relationship between these "measures" should be provided.

In the Cox model, the coefficients indicate the increase/decrease of log(hazard), while in the AFT model, the coefficients indicate the increase/decrease of log(time-to-event). From our understanding there is no exact relationship between the two measures. Both types of models can produce the estimates over the risk of developing the event over time. An AFT model factor that accelerates the occurrence of the event will increase the event risk; a Cox model factor that increases the hazard will increase the event of risk, but again there is no exact relationship between the two models regarding their risk estimates.

3) The non-linear fractional polynomial part is dealt with in twice half a sentence --- either this part is important enough to deserve some more details (then some more details on this should be provided) --- or it was just a "stump track" and should parenthetically be noted as such.

In order to keep this manuscript less statistically intensive, we have decided to remove this method from the manuscript (page 8 line 160-161; page 12 line 227-228) and doing so would not alter the results.

4) it is this reviewer's understanding that after model fitting, significant factors were aggregated through non-linear averaging (median) to produce a "predictive score" indicating low or high NI risk. This reviewer would appreciate some more details in the description, as this is a key contribution.

We have included in the supplementary material a detailed description on the calculation of the risk score (Supplementary Material page 3).

5) One of the presented results is that after risk classification, the high-risk patients exhibit twice as big empirical risk for NI compared to low-risk group. Immediately resulting thereof, the high-risk group accounts for a bit over 2/3s of the NI occurrences. Can the authors compare this figure against other models that are already "on the market"? (a random grouping into equally large groups would result in a roughly 50% split; how much "better than random" is the observed 70%?)

There are several publications reporting individual risk factors for postoperative NI. However, to the best of our knowledge, no existing risk prediction model or algorithm is available for us to compare with our model. There is one study using a similar scoring system to predict the overall hospital-acquired infections instead of postoperative infections (Chang Y-J et al. Predicting hospital-acquired infections by scoring system with simple parameters. doi: 10.1371/journal.pone.0023137).

6) Is there a compact way to graphically visualize the content of table 3 (and possibly appendix 2)? TR and HR CI, point estimates and p-values could be shown as "boxplot"-like bars relative to the neutral 1-axis. In this way, significant and large impacts will be more intuitively identifiable from the large crowd.

It is possible to exhibit the results with plots, but we would like to leave the table as it is: it might be slightly crowded but certainly provides more information than a plot.

7) Discussion: the results are presented as "delayed occurrence" versus "enhanced postoperative risk". Again, in the light of point 1 and 2, it is not entirely clear to this reviewer what is talked about: delayed occurrence of NI (or discharge?!)? How is post-operative risk quantified, here? (= accelerated occurrence of NI/discharge?) A priori delayed occurrence and risk are two different things that can not immediately be compared. The description should be a bit more verbose and complete, here.

Sorry for this confusion as a result of the switch between the two terms. In the revision, we have replaced “enhanced postoperative risk” with “accelerate the occurrence of postoperative NIs” (page 15 line 255-256).

We clarify here again that what we studied was occurrence of NIs. Please note that we were only interested in the time between operation and occurrence of postoperative NIs. Of course occurrence of NIs was very likely to delay the discharge, but the time span between NI diagnosis and discharge was beyond the scope of this work.

8) line 256: consisting => consistent

Corrected (page 15 line 262).

9) This reviewer is afraid that the list of possible (likely and relevant) confounders should be longer.

We agree that we have missed some potential risk confounders, such as mechanical ventilation, urinary catheterization, and some laboratory data (including serum albumin). However, we would like not to include these factors in the current analysis as their prevalence was low in our study population; besides, inclusion of more factors might not be appropriate given the relatively limited number of events.

10) Cross-validation: since the data are used twice---first to learn the AFT model parameters, second to stratify into risk-groups---it would be indicated to use separate parts of the data set for these tasks, to control "overfitting". How good is the risk-stratification outcome when applied on 50% of the data that was NOT used to fit the AFT model used to identify risk factors? The differences may be marginal, but it would be prudent to check.

In the revision, we reported the result of risk stratification based on the application of the reduced AFT model to an "independent" data set, which came from the same hospital but was established separately for this relatively "external" validation. Hopefully the reviewer would agree with us that this is appropriate. Corresponding changes have been made in the Statistical analysis section (page 8 line 173- page 9 line 179), Result section (page 14 line 233-241) and the Discussion section.

11) one in ten rule (and variants thereof): given 847 un-censored events how many predictor parameters can reasonably be expected to be fit? The authors might want to include a note on this.

The number of parameters that have to be estimated was 30 for the full AFT model and 24 for the reduced AFT model, and thus the number of events per parameter is 28 and 35, respectively, sufficiently higher than the one in ten rule.
